# Comparison of the effects of exercise with chondroitin sulfate on knee osteoarthritis in rabbits

**DOI:** 10.1186/s13018-018-0722-4

**Published:** 2018-01-22

**Authors:** Ning Ma, Tingting Wang, Lianyu Bie, Yang Zhao, Lidong Zhao, Shai Zhang, Li Gao, Jianhua Xiao

**Affiliations:** Key Laboratory of the Provincial Education Department of Heilongjiang for Common Animal Disease Prevention and Treatment, College of Veterinary Medicine, Northeast Agricultural University, Harbin, 150030 China

**Keywords:** Osteoarthritis, Exercise, Chondroitin sulfate, Rabbit

## Abstract

**Background:**

The aim of the study is to compare the effects of exercise therapy with chondroitin sulfate (CS) therapy in an experimental model of osteoarthritis (OA).

**Methods:**

Twenty-one New Zealand rabbits were randomly divided into four groups: normal group (N group, *n* = 3); OA control group (C group, *n* = 6); OA plus medication group (CS group, *n* = 6); and OA plus exercise group (E group, *n* = 6). Four weeks after modeling, the rabbits were subjected to exercise (artificial, 30 min/time, 4 times/week) or medicated with CS (2% CS, 0.3 ml/time, once/week) for 4 weeks. Histopathological changes in treated joints were examined after staining. X-ray and scanning electron microscopy was used to evaluate the different therapies by examining the surfaces and joint spaces of the articular cartilage. RT-qPCR was used to assess chondrogenic gene expression including Col2, Col10, mmp-13, il-1β, adamats-5, and acan in the experimental groups.

**Results:**

Histology showed both treatment groups resulted in cartilage that was in good condition, with increased numbers of chondrocytes, and the results of X-ray and scanning electron microscopy showed the therapeutic effect of exercise therapy is equivalent to CS therapy, surface articular cartilage was flat, and the of cartilage layer was thinning. All treated groups induced the expression of Col10 and Col2 and decreased expression of mmp-13, il-1β, and adamats-5 compared with the control groups. The expression of acan was upregulated in the E group and downregulated in the CS group. Furthermore, expression of Col10 was higher and il-1β was lower in the exercise group compared to that of the CS group.

**Conclusion:**

These results indicate that exercise has a positive effect on OA compare with CS, and it also supplies reference for the movement mode to improve function.

## Background

Osteoarthritis (OA), one of the most common chronic conditions, is a chronic degenerative joint disease characterized by cartilage degeneration and erosion, fibrosis, and osteophyte formation, leading to pain and disability in the worst cases [1].

The early changes in articular cartilage in OA is characterized by the loss of proteoglycan and a reduction in the expression of collagen 2 (Col2) gene without any changes to the regularity of the structure the articular tissue [2]. In contrast, the expression of PCNA, Ki67, and matrix metalloproteinase 13 (mmp-13) are significantly increased [3–5], in addition to observations of chondrocyte changes similar to the stages of cell hypertrophy [6, 7]. Severe OA mainly manifests as cartilage surface cracks or fibrosis and defects in the cartilage with the banded structure in disorder [8]. There are cracks in the fibrotic area, extending to the calcified zone accompanied by severe loss of proteoglycan and degradation of Col2 [9], and upregulation of the expression of mmp-13 and mmp-2 [10]. The expression of genes related to the terminal differentiation of chondrocytes has been found in cell clusters around the cracks, such as Col2, collagen 10 (Col10), and TGF-β3 [11].

There are many ways to treat OA including slimming in overweight patients, medication, non-pharmacological interventions, and surgery. During the last 15 years, the focus of treatment has begun to shift to non-medicine treatment. It is generally believed that exercise can reduce joint pain and improve joint function. Latham and Liu suggest exercise can strengthen the quadriceps [12], which effectively reduces lower limb pain and improve joint function [13], and Bennell et al. believe the key to the treatment of OA is restoring joint function [14, 15]. Iijima et al. studied moderate exercise can also prevent OA from causing joint tissue damage [16]. Chondroitin sulfate (CS), a slow-acting drug for the treatment of arthritis, has an anti-inflammatory effect by reducing the synthesis of proteolytic and inflammation-related enzymes, and proinflammatory cytokines [17, 18]. In 2010, the International Society of OA pointed out that CS not only reduces the symptoms of OA but also modifies the structure of the cartilage after prolonged injection, so that it is more advantageous in the therapy of OA [19].

This paper studies whether exercise has some stimulation on cartilage excluding the effect of muscular changes, weight, joint loading, and so on; leads to some changes in the surface of cartilage such as collagen, polysaccharide, and chondrocytes; and ultimately achieves the purpose of treatment of arthritis. CS appears to act as a protective agent of cartilage in the treatment of OA, so it can be used as a positive control group, to study the effect of exercise on the treatment of arthritis using clinical, histological, and genetic techniques.

## Methods

### Animals and surgery

New Zealand rabbits (*n* = 21) with a body weight in the range 2.5 ± 0.5 kg were used in the study. The rabbits fasted for 12 h before surgery and were then anesthetized with ether. They were randomly divided into four groups. Except for the normal group (N, *n* = 3), rabbits were made osteoarthritic as per the Hulth-Telhag model [20]; as described before, the medial side of the joint capsule was opened without cutting the articular surface and the anterior cruciate ligament was cut and a portion of the meniscus removed, under sterile conditions. The normal group had a sham operation. Penicillin and streptomycin (Yocon Biotechnology Co., Ltd., China) were administered twice daily for 3 days post-operatively to avoid surgery-related infection. After 4 weeks, the rabbits were randomly assigned to the various groups.

### Treatment

Rabbits were randomly divided into three groups after the arthritis models were successfully established. Rabbits in the 2% CS group (CS, *n* = 6, 30 min/time) were injected with 0.3 ml CS (Biosharp, China) once per week. In the exercise group (E, *n* = 6), put the electric animal treadmill upside down and the rabbits were lying on the board, make it passively exercised for 30 min, four times a week. The control group (C, *n* = 6, saline) animals served as a control and were maintained as an osteoarthritic control and not given any treatment.

### Histology

After 34 days of treatment, the articular cartilage from the middle part of the load-bearing region was shaved off with a scalpel. Sections were frozen and stored in liquid nitrogen if scheduling so required. Some pieces were fixed in 10% formaldehyde solution then embedded in paraffin, then finally stained with hematoxylin eosin (HE), toluidine blue, and alcan blue and prepared for light microscopy according to standard procedures.

### X-ray assessment

Anterioposterior and left-lateral images were performed on the knee joints of animals after 34 days of treatment, using X-rays and direct digital radiography (DR), to estimate the degree of cartilage degradation and joint space reduction under anesthesia. Exposure to X-ray was conducted at 300 MA, 50 kV with a 0.03-s exposure time, and a 100-cm tube to film distance for AP projection.

### Scanning electron microscopy

After sacrifice, the knees of the hind legs of the animals were dissected in sterile conditions. Medial femur tissue measuring 0.4 mm × 0.4 mm × 0.2 mm was harvested, submerged in a sterile solution of 0.9% NaCl at room temperature to prevent oxidation. All three groups were fixed in 4% paraformaldehyde for 2 days then submerged in OsO_4_ for 2 h. The samples were transferred to a critical point dryer then coated with gold to generate an 18-nm-thick coating. Changes in the microstructure were determined using scanning electron microscopy (SEM).

### RT-qPCR analysis

Total RNA was extracted with Trizol® Reagent (TransGen Biotech, Beijing, China) according to the manufacturer’s instructions. Reverse transcription was performed using  PrimeScript^TM^ RT Master Mix (Takara) according to the manufacturer’s instructions. The resultant cDNA was used to amplify Col2, Col10, acan, mmp-13, interleukin-1β (IL-1β), and ADAM metallopeptidase with thrombospondin type 5 motif (admats-5) transcription. Primers were synthesized by Comate Bioscience Company Limited (Beijing, China) and are shown in Table 1. Quantitative reverse transcription polymerase chain reaction (RT-qPCR) amplification was carried out using SYBR green master mix (Toyobo) in a LightCycler 2.0 (Toyobo) using the following conditions: pre-incubation at 95 °C for 30 s; 45 cycles of amplification with denaturation at 95 °C for 5 s, annealing at 57 °C for 20s, and extension at 72 °C for 20 s; and 1 cycle of melt curves at 95 °C for 0 s, 65 °C for 15 s. Finally, a cooling step was performed at 40 °C for 30 s. All samples were performed in triplicate. After each reaction, the number of cycles required for the threshold (Ct) value to be exceeded was recorded, reflecting exponential kinetic measurements.

**Table 1:** Transcripts and sequences of each primer used in RT-qPCR

Gene	NCBI number	Forward Primer, 5′–3′	Reverse Primer, 5′–3′
GAPDH	KJ_875954.1	CCCTCAATGACCACTTTGTG	GGTTTGAGGGCTCTTACT CCT
Col2	S_83370	GCACCCATGGACATTGGAGG	AGCCCCGCACGGTCTTGCTT
Col10	XM_002714724	GAAAACCAGGCTATGGAACC	GCTCCTGTAAGTCCCTGTTGTC
MMP-13	NM_1082037	TTCGAGTCATGCCACAAAT	TAAGCTTTGCCCTGAAACCT
IL-1β	NM_001082201	TGACGGCCTGAGAACTTTCT	CATACGTGCCAGACAACACC
ADAMTS-5	AF_247708	CAGTGTTCTCGCTCTTGTGG	CTGGGTGCAGGGTTATTGC
Aggrecan	L_38480	ATGGCTTCCACCAGTGCG	CGGATGCCGTAGGTTCTCA

*Col2* collagen 2, *mmp-13* matrix metalloproteinase 13, *Col10* collagen 10, *IL-1β* interleukin-1β, *ADAMTS-5* ADAM metallopeptidase with thrombospondin type 5 motif

### Data analysis

The 2^-ΔΔCt^ method was adopted to calculate relative mRNA concentrations against glyceraldehyde-3-phosphate dehydrogenase (GAPDH) as the reference gene [21]. All data were statistically analyzed using Graph Pad Prism v7.0 software (Graph Pad Software, Inc., La Jolla, CA, USA). Data are expressed as mean ± standard deviation (SD). Analysis of significance was calculated using two-way analysis of variance (ANOVA). Differences having *p* < 0.01 were regarded as very significant, and results were considered statistically significant in all analyses at *p* < 0.05.

## Results

Two rabbits were randomly selected from the experimental groups for X-ray photography using the same conditions. The articular cavity was observed narrowing in comparison with normal knees, proving that the experimental model of OA was successful.

### Assessment of tissue morphology and histology

**Fig. 1:**
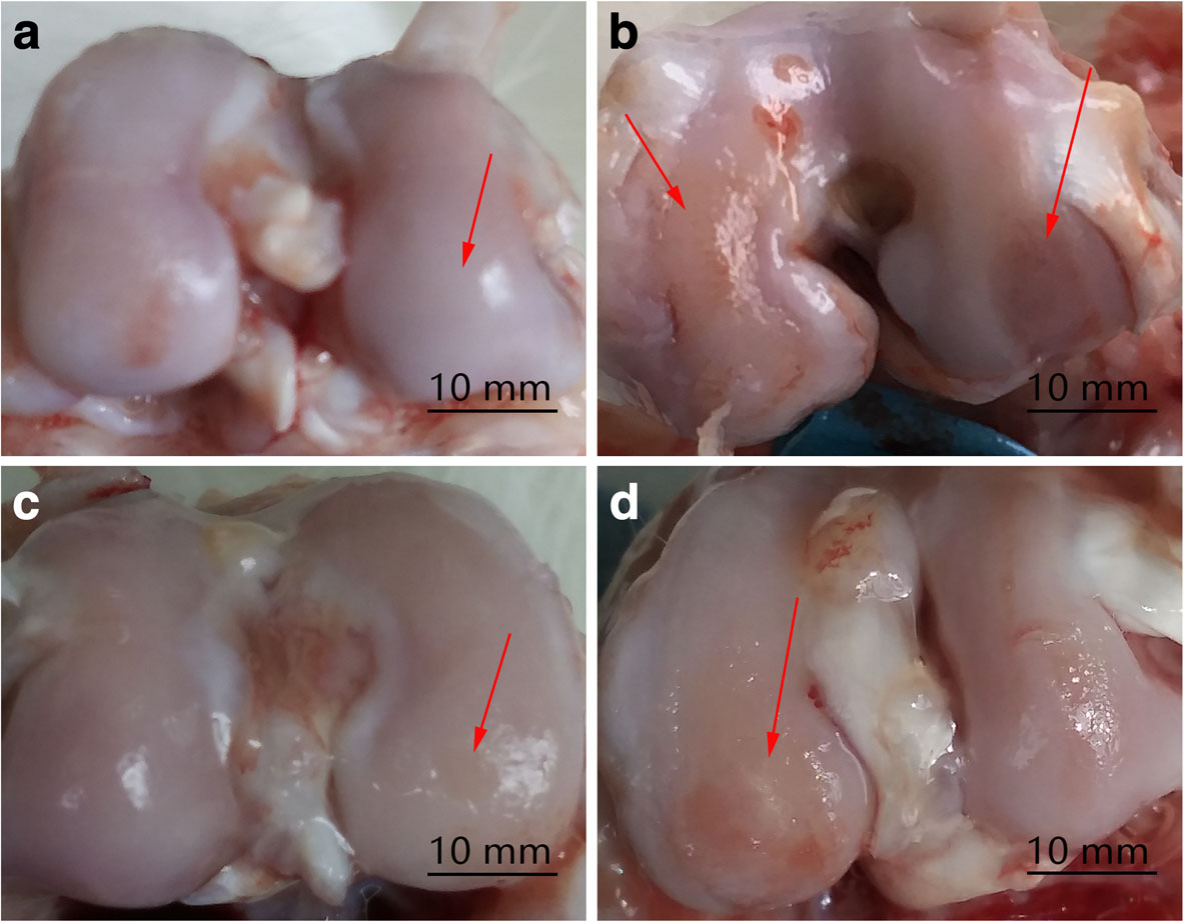
Macroscopic view of rabbit articular cartilage. **a** The femoral condyles of the Normal group knee joints had no deterioration in the cartilage. **b** Control group showed obvious pathological changes including cartilage proliferation, exposure of subchondral bone, and cartilage hyperplasia. **c** CS group had a relatively uniform area with some small craters and the appearance of cracks. **d** The performance characteristics of the E group are erosion of the cartilage and swelling of the femoral condyles

**Fig. 2:**
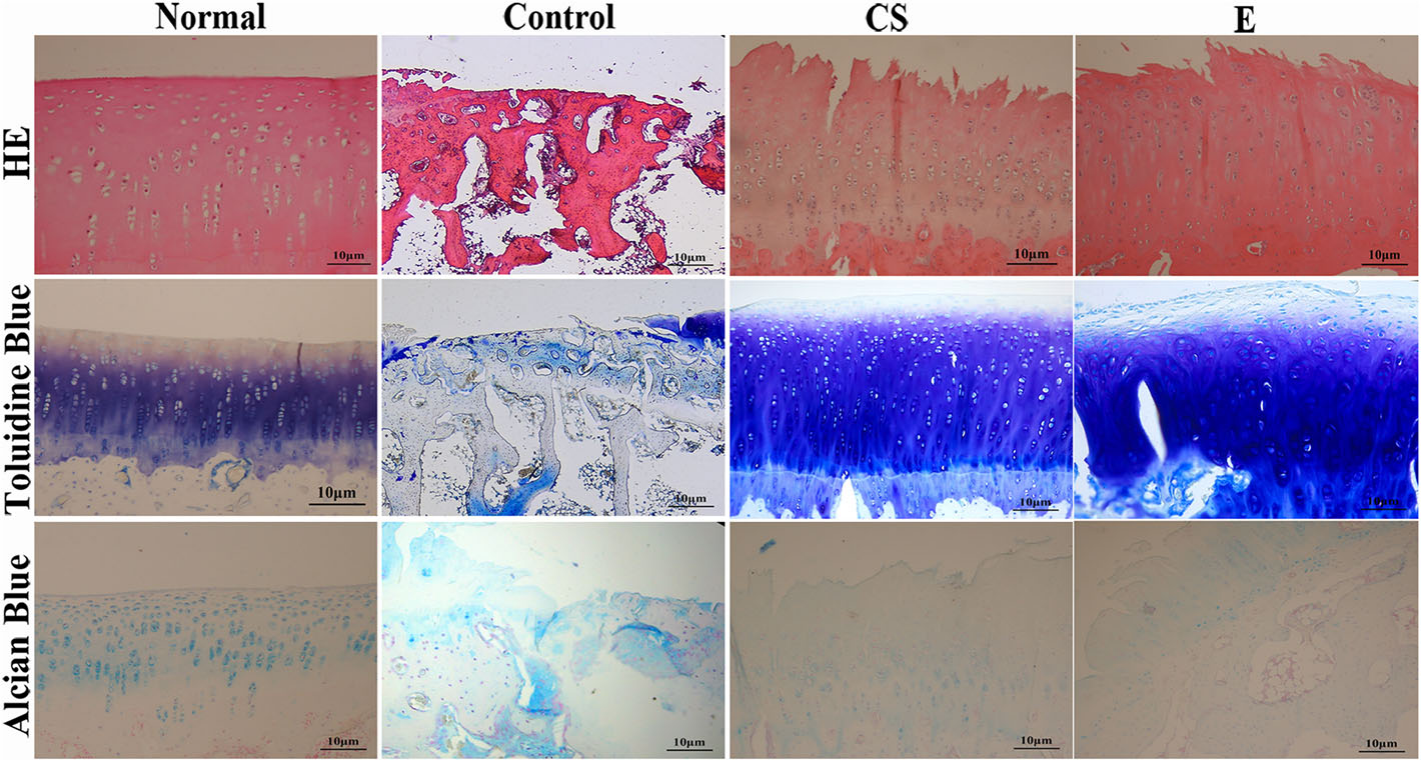
Histological analysis of the articular cartilage

In the healthy rabbit knees, all the joints contained healthy and smooth articular cartilage using a visible check by naked eye. Obvious morphological differences were observed in the CS group and exercise group compared to the controls. Compared with the N group, articular cartilage hyperplasia, cartilage surface roughness, and obvious cracks were observed in all the experimental groups, in addition to a thinned cartilage layer (Fig. 1), resulting in an alteration to the histology, consistent with these morphological changes. Normal chondrocytes secrete cartilage matrix that principally comprises collagen II fibers and proteoglycans. In the event of chondrocyte degeneration or necrosis, they either secrete abnormal proteoglycan or no longer secrete any at all, as evidenced by loss of toluidine blue staining or uneven dyeing [22, 23]. HE, alcian blue and toluidine blue staining were conducted to observe tissue histomorphology (Fig. 2). The normal cartilage matrix was consistently colored, chondrocytes were arranged in order, and the tidal lines were intact. The cartilage in the control group was markedly thinned and the subchondral bone slightly enlarged. It can be seen from the toluidine blue staining that the cartilage surface was smooth in the CS and E groups with partial destaining and tidal lines that were clear compared with those of the control group. The number of cartilage cells in the E and CS groups was significantly larger than in the C group, as was the thickness of cartilage layer. HE staining showed that the surface of the cartilage in the exercise and CS groups was irregular, and the chondrocytes were disordered and showed evidence of mitosis and proliferation.

**Fig. 3:**
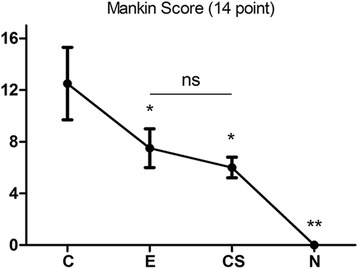
Mankin scale for joint destruction (a–d). Data are means ± SD of three rabbits for each group. *p < 0.05 and **p < 0.01 versus untreated control, n.s. not significant

The severity of histological changes in the tissue was evaluated using Mankin scores [24]. According to the scoring, differences between the CS and E groups were not clear. Mean scores for the control, CS, exercise, and normal groups were 12.5 ± 2.8, 6.0 ± 0.8, 7.5 ± 1.5, and 0.0 ± 0.0 at 34 days, respectively (Fig. 3), indicating that severe-to-mild changes representing OA were observed, consistent with the histological changes.

### Scanning electron microscopy and radiography of the surface of articular cartilage

**Fig. 4:**
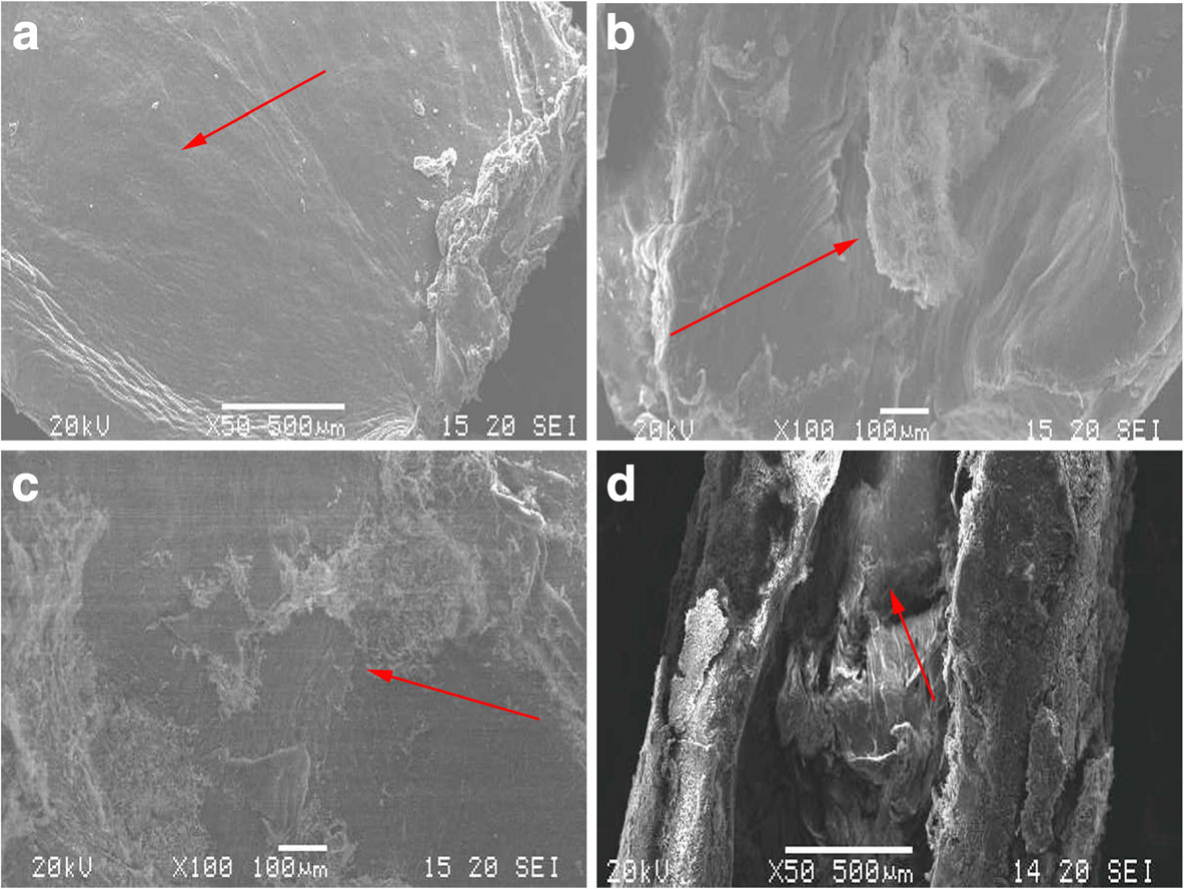
Scanning electron microscopy view of surface of fresh cartilage. **a** Normal group was uniform without splits or lacunae. **b** Control group had deep splits on the surface. **c** Cartilage from rabbits where chondroitin sulfate (CS) had been administered were relatively uniform, with a visibly twisted wrinkled texture in other areas. **d** Rabbits that had exercised (E group) had a relatively rough surface

**Fig. 5:**
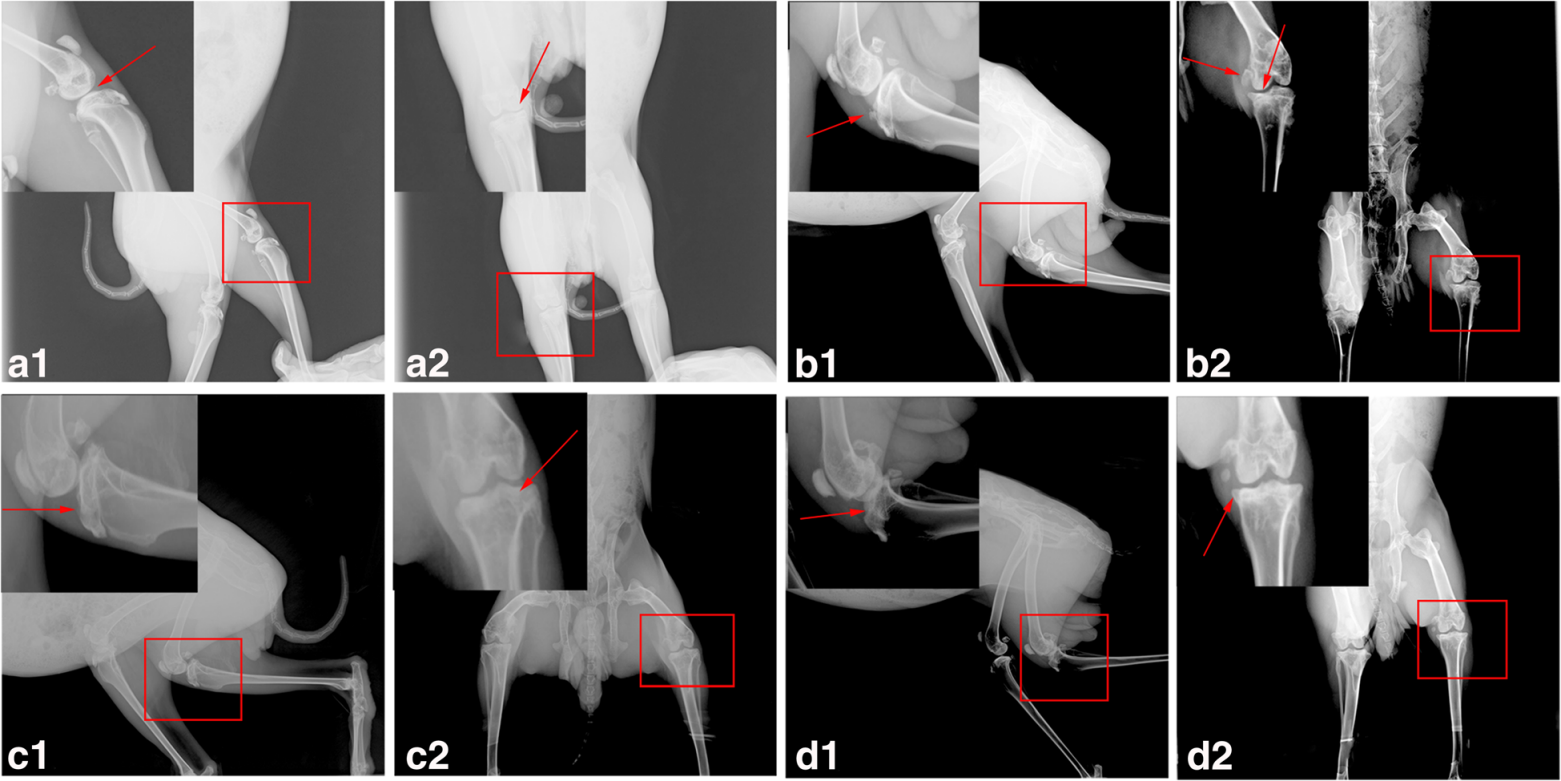
Lateral and craniocaudal radiographs from the joints of rabbits

The macroscopical and microscopical assessment of the knee joints showed multiple states. Normal articular cartilage exhibited a uniform area without splits, lacunae, or cartilage proliferation and was investigated using scanning electron microscopy (Fig. 4a). According to X-ray analysis, the surface was smooth and continuous (Fig. 5a). Those in the control group were significantly deteriorated with a rough appearance where the surface had fissures that had widened and collagen fibers that were exposed, loose, broken, and turned upwards (Fig. 4b). As shown in Fig. 5b, formation of cartilage proliferation and cartilage surface defects and thinning of the cartilage layer can be seen indirectly from the radiograph, with spacing becoming larger. In the CS group, the articular cartilage surface was no longer flat, with a visibly twisted wrinkled texture and a slightly concave shape (Fig. 4c). Radiographic images of the joints in the CS group showed that they had attachments of low density material (Fig. 5c). In the E group, the collagen fibers were completely exposed and broken giving the appearance of a rough cartilage surface (Fig. 4d). Small osteophytes had formed on the surface of the articular cartilage, and the widened space of the joint had improved after treatment with CS or exercise (Fig. 5c, d).

### MMP-13, Acan, Col2, Col10, ADAMTS-5, and IL-1β mRNA expression

**Fig. 6:**
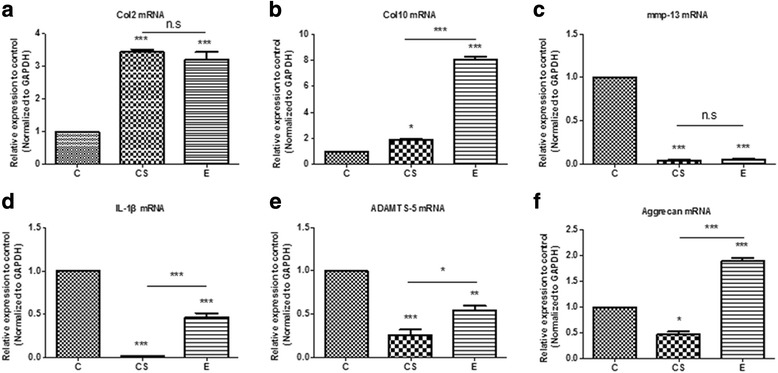
Gene Expression. RT-qPCR was used to examine the mRNA expression of **a** Col2, **b** Col10, **c** mmp-13, **d** il-1, **e** adamts-5, and **f** acan genes in each group. The results showed no evident differences between the CS and E groups for the genes **a** Col2 and **c** mmp-13. The expression of **c** mmp-13, **d** il-1, and **e** adamts-5 genes in the treated groups were decreased compared to that of the control group. The expression of **f** acan and **a** Col2 genes in the E group increased, while the expression of **f** acan decreased in the CS group. *p < 0.05, **p < 0.01, ***p < 0.001 indicate significant differences between groups. n.s. not significant. The 2-ΔΔCt method was adopted with GAPDH as the reference gene

As shown in Fig. 3, the expression of Col2 mRNA in the cartilage of E and CS groups increased substantially compared with the control group (Fig. 6a). In addition, the chondrocytes exhibited a noticeable upregulation of the mRNA expression of Col10 in the CS and E groups and compared with the CS group; the level of Col10 increased significantly in the E group (Fig. 6b). The expression of mmp-13 and il-1β in the E and CS groups decreased substantially compared with the control group (Figs. 6c, d). Similarly, the expression of adamts-5 in the CS group decreased significantly and lowered in the E group (Fig. 6e). However, treatment of exercise observably increased the mRNA of acan in contrast to the CS group (Fig. 6f).

## Discussion

OA is one of the most common joint diseases affecting human health, ultimately a degenerative disease of the joints, without racial and regional differences [25]. Our findings verified that in the control group. the surface of the cartilage was not smooth and was accompanied by destruction of the articular cartilage, cartilage fibrosis, osteophyte formation, synovial proliferation, and loss of joint function leading to pain and potential disability (Fig. 1).

In recent years, CS has been listed as a daily-use drug to improve the symptoms of OA. The European League against Rheumatism has suggested that CS is an effective drug for the treatment of knee OA [4]. As there are no blood vessels within articular cartilage, relying on synovial fluid to provide nutrition, CS can be used as a delivery pipeline, transporting nutrients to the joints, allowing an increase in joint metabolism and inhibition of the pain of OA [26–29]. The cartilage surface in the CS group was smooth but with a visible distortion in the texture and a slight breakage of collagenous and elastic fibers compared with the control group (Fig. 4c). Jr. et al. suggest CS allows direct mechanical and frictional forces to be buffered and traps water molecules within its chains [30]. Our results showed that the drug group alleviated the pathological changes to OA under the action of CS. In this experiment, intumescentia of the articular surface is too small to be seen with the naked eye compared with the control group (Fig. 1c). The articular cartilage was slightly discolored in the CS group, with a surface roughness that was not clearly obvious. Histology revealed that there were biological growths on the cartilage surface in addition to a significant increase in the number of chondrocytes and quantity of matrix, constituting a significant increase in the number of each cartilage unit (Fig. 2). CS is an important component of articular cartilage. Mice that are knockout for the N-acetylgalactosyltransferase-1 gene, an important enzyme used in the synthesis of CS, have some characteristics similar to those suffering from OA, including a reduction in proteoglycan molecules, the rapid catabolism of acan and disordered Col2 fibers [31]. Compared with the control group, the expression of Col2 (*p* < 0.01) and Col10 (*p* < 0.05) mRNA increased, while acan decreased (*P* < 0.05). CS exerts its anti-inflammatory effects through the inhibition of reactive oxygen species activity or the activation of protein kinases, reducing the synthesis of proteolytic enzymes (mmp-3, mmp-9, and mmp-13), inflammation-related enzymes (pla2, cox-2, nos-2), and proinflammatory factors (il-1β and tnf-α) [6, 7]. In this study, administration of CS reduced the expression of mRNA for mmp-13, il-1, and adamts-5 (*p* < 0.01), which concurs with previous [32] studies.

At present, exercise as a convenient and cheap treatment of OA has been investigated in recent years. The previous study revealed that exercise had an effect on bone stabilization of the OA joint [33]. Exercise intervention for strength training does not provide superior outcomes in pain or disability [34]; exercise therapy does not slow down the symptoms of arthritis by enhancing muscle strength or reducing weight on the joint. In this experiment, after the success of arthritis modeling, the rabbits were subjected to exercise therapy for a period of 4 weeks, once every 2 days. Compared with the control group, the joint swelling was not apparent (Fig. 1d). And the X-ray structures revealed that thickness of articular cartilage has been improved compared with the control group (Fig. 5d), which was consistent with the research of Kiviranta et al. [35]. Chondrocytes are the key to maintaining healthy cartilage extracellular matrix [36]; in Fig. 5 the number of cells in each cartilage unit comprising cartilage and chondrocytes increased significantly. The exercise stimulates the proliferation of chondrocytes, and then achieves the purpose of restoration.

The level of matrix collagenase in OA was increased, and the expression of MMPs and adamts-5 were higher in the deteriorated matrix, while expression of Col2 and acan were exhibited [35]. There was a high concentration of Col2 in the cartilage matrix amongst collagen fibers. The dynamic balance of Col2 and proteoglycan production and consumption is the direct cause of the loss of biomechanical properties in articular cartilage. In the case of inflammatory infiltration in articular cartilage, chondrocytes lose their normal phenotype and convert into mast cell to release Col10. In the exercise group, the relative expression of Col2 and Col10 genes increased compared to that in the control group (*p* < 0.01), and the relative expression of Col10 mRNA increased (*p* < 0.01) in the CS group (Fig. 6a, b). The MMP and ADAMTS families have been shown to be the two major types of enzymes that remove cartilage extracellular matrix, which is composed of proteoglycan and collagen. MMP-13 acts mainly on cartilage collagen, so its inhibition would inhibit the degradation of Col2, preserving the cartilage extracellular matrix structure [37]. Similarly, in Fig. 6c, the mRNA levels of mmp-13 decreased significantly (*p* < 0.05) in the exercise group compared with those in the control group; it would reduce morphological changes in the cartilage and slow down the process of OA. However, there was no significant difference in the expression of mmp-13 between the exercise and CS groups. ADAMTS-5 mainly decomposes acan, the degradation of which is prevented after knockout of the adamts-5 gene, reducing the pathological process of OA [38, 39]. In the CS group, the relative expression of the acan gene increased compared with the control group (*p* < 0.01), while the mRNA levels of adamts-5 decreased significantly (*p* < 0.05). IL-1β can affect the activity of chondrocytes through the synthesis of matrix metalloproteinases and plays a key role in blocking the synthesis of extracellular matrix Col2 and proteoglycans in chondrocytes [40]. Exercise training effectively regulates the inflammatory process caused by knee OA [41]. We observed a decrease in the il-1β mRNA levels (*p* < 0.05) in the exercise group (Fig. 6d). This study was consistent with the conclusion by Yang Y et al., and they corroborated that moderate treadmill exercise can alleviate the severity of cartilage lesions in experimental OA through its anti-inflammatory activity of LXA4 and the NF-κB pathway [42]. The surface of the articular cartilage had small osteophyte formations in the exercise group but not the CS group (Fig. 5c, d). The cartilage in the CS group was cracked and the number of cartilage units had decreased. Nevertheless, the crevices in the exercise group cartilage were smaller and the number of cartilage cells significantly increased.

Our findings suggest that exercise therapy can improve the metabolism of chondrocytes by promoting the permeation and diffusion of synovial fluid to the articular cartilage due to joint movement and promote regeneration of cartilage tissue by increasing its nutrition metabolism. In accelerated repair of the injured joint and tissue surrounding the articular cartilage, an exercising joint can stimulate the proliferation of chondrocytes, which is conducive to the transformation of undifferentiated stem cells into chondrocytes to fulfil the purpose of repairing articular cartilage and provide relief of injury of the joint. The result of this experiment implies a possibility that exercise not only avoids drug metabolism but also improves the symptoms of OA. Using exercise, CS can be replaced in the treatment of OA in some respects.

## Conclusions

In conclusion, we have demonstrated that exercise has a good therapeutic effect on OA equate to CS. The limitations of this study should focus on changing the way of movement to have an effect on the cartilage and not only changing gait biomechanics to reduce pain and improve physical function.

## Abbreviations

ADAMTS-5: ADAM metallopeptidase with thrombospondin type 5 motif
Col10: Collagen 10
Col2: Collagen 2
CS: Chondroitin sulfate
Ct: Cycles required for the threshold
DR: Direct digital radiography
GAPDH: Glyceraldehyde-3-phosphate dehydrogenase
HE: Hematoxylin and eosin stain
IL-1β: Interleukin-1β
mmp-13: Matrix metalloproteinase 13
OA: Osteoarthritis
RT-qPCR: Reverse transcription and quantitative polymerase chain reaction
SD: Mean ± standard deviation
SEM: Scanning electron microscopy
